# Highlights from the 14^th^ St Gallen International Breast Cancer Conference 2015 in Vienna: Dealing with classification, prognostication, and prediction refinement to personalize the treatment of patients with early breast cancer

**DOI:** 10.3332/ecancer.2015.518

**Published:** 2015-03-31

**Authors:** Angela Esposito, Carmen Criscitiello, Giuseppe Curigliano

**Affiliations:** Division of Experimental Cancer Medicine, Division of Radiotherapy and Division of Pathology, European Institute of Oncology, Milan, Italy

**Keywords:** 14th St Gallen Consensus Conference 2015, early breast cancer

## Abstract

The refinement of the classification, the risk of relapse and the prediction of response to multidisciplinary treatment for early breast cancer has been the major theme of the 14^th^ St Gallen International Breast Cancer Consensus Conference 2015. The meeting, held in Vienna, assembled 3500–4000 participants from 134 countries worldwide. It culminated, on the final day, with the International Consensus Session, delivered by 40–50 of the world’s most experienced opinion leaders in the field of breast cancer treatment. The panelist addressed the “semantic” classification of breast cancer subtypes by pathology-based biomarkers (e.g. estrogen receptor, progesterone receptor and HER2) vs genomic classifiers. They also refined the biomarker prognostication dissecting the impact of the various gene signatures and pathologic variables in predicting the outcome of patients with early breast cancer in terms of early and late relapse. Finally they addressed the challenges stemming from the intra- and inter-observer variability in the assessment of pathologic variables and the role of gene signatures for the prediction of response to specific therapeutic approach such as endocrine therapy and chemotherapy and for personalizing local treatment of patients with early breast cancer. The vast majority of the questions asked during the consensus were about controversial issues. The opinion of the panel members has been used to implement guidance for treatment choice. This is the unique feature of the St. Gallen Consensus, ensuring that the resulting recommendations will take due cognizance of the variable resource limitations in different countries. Information derived from evidence based medicine and large meta-analyses is of obvious and enormous value. The weakness of this approach is that it gives particular weight to older trials (which have accumulated more event endpoints) and is frequently unable to collect sufficient detail on the patients and tumors in the trials to allow assessment of whether the treatments which are better on average offer equal value to all currently definable patient subgroups. What St Gallen can provide is clinically useful updated breast cancer treatment consensus for the majority of patients treated outside of clinical trials (>90%) in most countries.

## Conference Report

The primary aim of the 2015 St Gallen Consensus conference was to provide rational recommendations for personalizing the approach to the treatment of women with early breast cancer. Major issues that have been highlighted included: 1) Clinical utility of genetic tools in terms of prognostic assessment and prediction of response; 2) Tumor extent to estimate level of benefit justifying treatment for the individual patient; 3) Estimates of the risks of therapy and patient preference to define preferred management. The areas of controversy discussed during the meeting were: 1) Surgery of the primary; 2) Surgery of the axilla; 3) Radiation: partial breast, post-mastectomy, nodal areas, advanced technologies; 4) Pathology; 5) Multi-gene signatures; 6) Endocrine therapies in pre- and post-menopausal setting; 7) Duration of endocrine therapy; Chemotherapies: indication in intrinsic subtypes, duration, regimens; 8) Anti-HER2 therapies: combination, duration; 9) Neo-adjuvant systemic therapy; 10) Bisphosphonates: anti-tumor effects; 11) Treatment in special populations (elderly, frail, young women, male patients); 12) Role of diet and exercise. Here we present a conference report that will highlight some of the controversial areas discussed during this outstanding meeting.

## News since St Gallen 2013

### Surgery of breast cancer

Dr Monica Morrow from New York, USA, opened the scientific session with an outstanding and comprehensive talk on the surgical management of early breast cancer. She stated that it is increasingly clear that local control is a function of disease burden, tumor biology, and use of effective systemic therapy, offering the opportunity to decrease the extent of surgery and reduce the burden of treatment in some patients. Changes in practice reflecting this understanding have already occurred and include new guidelines on margin width in breastconserving therapy (BCT) and alternatives to axillary dissection (ALND). The ACOSOG Z0011 study was a landmark trial demonstrating that ALND is not necessary in cN0 women undergoing BCT with metastases to 1–2 sentinel nodes (SNs) [1]. The AMAROS trial studied axillary nodal RT as an alternative to ALND in patients with positive SNs and observed no differences in axillary recurrence or survival compared to ALND [2]. The fact that microscopic disease in the axilla is controlled with systemic therapy and less than full dose RT, coupled with the observation that locoregional recurrence (LRR) as a proportion of all recurrences decreased from 30% to 15% between 1985 and 2010, prompted the Society of Surgical Oncology (SSO) and the American Society for Therapeutic Radiation Oncology (ASTRO) to develop a multidisciplinary consensus on the optimal margin width in BCT [3]. Based on a metaanalysis and other literature, it was concluded that margins more widely clear than no ink on tumor do not reduce local recurrence, and the routine use of re-excision to obtain larger margins is not indicated. A major issue for the future is how to resolve several divergent viewpoints. Studies using neoadjuvant chemotherapy (NAC) will offer important information.

### Clinical utility of genetic signatures

Dr Dan Hayes reported on the clinical utility of genetic signatures dealing with risk prediction of early vs late recurrences. Adjuvant endocrine therapy (ET) reduces the risk of distant recurrence and mortality in women with hormone receptor (HR) positive early stage breast cancer. These patients have ongoing recurrences over many years at long term follow up. Extended adjuvant ET reduces recurrence. ET induces side effects such as hot flashes and sexual dysfunction that will impact on quality of life. Accurate assessment of the risk of late recurrence would permit appropriate extended ET decisions. Several multiparameter assays [IHC4, 21-gene OncotypeDX, 12-gene Endopredict, PAM50, 2-gene Breast Cancer Index (BCI)] have been investigated. The clinical validity of IHC4, OncotypeDX, and the BCI assays have been compared in the ATAC trial. All three assays had significant prognostic ability for early distant recurrence, but BCI-L was the only assay that predicted recurrence beyond 5 years [4]. The risk of distant relapse for patients with intermediate or high risk BCI-L scores exceed 10% during years 5–10, while that for patients with low risk score was approximately 3%. These data suggest that these assays have “clinical validity” for selecting women with ER positive breast cancer who might be spared extended ET.

### Treatment of pre-menopausal women

Dr Marco Colleoni addressed the topic on endocrine adjuvant treatment in pre-menopausal women. Endocrine treatments continue to represent a crucial component of adjuvant therapies for pre-menopausal patients with tumors that express steroid hormone receptors (HR). Endocrine agents are commonly well tolerated. Nevertheless selected side effects should be considered when evaluating treatment options, based upon risk of relapse, degree of endocrine responsiveness, expectations of the patient and co-morbidities. Tamoxifen (T) should still be regarded as a proper endocrine therapy in a large number of pre-menopausal patients. However, according to the results of the SOFT and TEXT trials, the use of T alone in selected higher risk patients may be questioned. The SOFT and TEXT trials, dedicated to pre-menopausal women with HR+ breast cancer, were developed to determine the role of ovarian function suppression (OFS) in women who remain pre-menopausal and are treated with T alone (OFS question) and also to test whether adjuvant aromatase inhibitor (AI) improves outcomes in patients treated with OFS (AI question) [5, 6]. Overall, in the SOFT study patients did not benefit from the addition of OFS [5]. Nevertheless, for women at higher risk of recurrence who received adjuvant chemotherapy and maintained pre-menopausal levels of estradiol, addition of OFS to tamoxifen reduced the risk of recurrence. The magnitude of the effect was larger in younger patients [5]. In the TEXT trial, adjuvant treatment with exemestane plus OFS, as compared with T plus OFS, significantly improved disease-free survival, breast cancer-free interval and distant disease-free survival, therefore representing a new treatment option [6]. Tailored endocrine treatments should be considered in pre-menopausal patients with endocrine responsive tumors. In fact, breast cancer in pre-menopausal patients includes heterogeneous groups of tumors and patients where issues of safety, quality of life and subjective side-effects should be properly weighted with the benefit of adjuvant endocrine therapy [6]. Patient preferences should be regularly taken into consideration in the definition of the threshold of expected benefit at which therapies should be attempted.

### Role of large randomized trials and Big Data

Dr Clifford Hudis discussed the role of prospective randomized trials in the era of precision medicine. A distinguishing feature of medical oncology in general, and breast cancer treatment in particular, has been a longstanding reliance on, and trust in, prospective randomized clinical trials as the basis of recommendations for standard therapy. Large randomized trials with long follow-up were needed for similar reasons: to detect modest differences in the risks of relapse and death over many years. This culminated in the performance of metaanalyses by the Early Breast Cancer Trialists’ Collaborative Group that led to broadly accepted standards of care such as tamoxifen for hormone-receptor positive disease and chemotherapy for higher risk subsets. Changes in our understanding of the molecular biology of cancer will challenge our historical approach. Breast cancer is not a single disease and molecular segmentation deriving from molecucal biology will change the scenario of drug design. The relatively rapid uptake of electronic medical records (EMRs) in many communities and especially in oncology may provide an alternative path to the development of useful and reliable evidence. EMRs should be able to link tumor genotypes, patient phenotypes, and routine treatment choices with outcomes. This may illuminate trends that can guide prospective research while also perhaps informing routine care. This approach may allow us to conduct more efficient drug development in larger numbers of patients despite the rapid growth in our perception of the complexity of breast and other cancers.

## Biology of Breast Cancer

Three sessions were dedicated to the biology of breast cancer. The major topics addressed were role of genetic assay in breast cancer classification and treatment, tumor heterogeneity and role of immune system and microenvironment. Gene-expression profiling has had a considerable impact on our understanding of breast cancer biology. During the last 15 years (yrs), 5 intrinsic molecular subtypes of breast cancer (Luminal A, Luminal B, HER2-enriched, Basal-like and Claudin-low) and a normal breast-like group have been identified and intensively studied [7]. Within hormone receptor (HR)-positive and HER2-negative breast cancer, the Luminal A and B subtypes represent the vast majority of cases. Compared to Luminal A tumours, Luminal B tumours are characterized by higher expression of proliferation/ cell cycle-related genes and lower expression of several luminal-related genes such as the progesterone receptor (PR) [7]. Clinically, Luminal B tumours show higher pathological complete response rates following neoadjuvant multi-agent chemotherapy but worse distant recurrence-free survival at 5- and 10-yrs regardless of adjuvant systemic therapy compared to Luminal A tumours. This Luminal A vs B classification, together with tumour size and nodal status, also predicts distant recurrence within the 5- to 10-yrs of follow-up and thus may inform decisions concerning the length of endocrine therapy treatments (i.e. 5 vs 10 yrs). Interestingly, although we and others have proposed pathology-based surrogate definitions of the Luminal A and B subtypes using semi-quantitative IHC scoring of Ki-67 and PR, the discordance rate versus multi-gene expression assays is still high (~30–40%) [8]. The recent technological advances and the extraordinary team efforts of consortia like the Cancer Genome Atlas (TCGA) and the Molecular Taxonomy of Breast cancer International Consortium (METABRIC) have dramatically increased our understanding of the molecular pathways and their derangements in human solid tumours [9]. The combined evaluation of recurrent genomic abnormalities (gene mutations and gene copy number variations) and transcriptomic profiles has led to a continuous refinement of the molecular classification of breast cancer with prognostic implications. The molecular stratification of Luminal A tumours allows the identification of new subgroups with a significantly worse prognosis, and possibly the ability to predict resistance to endocrine therapy. It remains to be assessed when and how much this more comprehensive understanding of the molecular heterogeneity of breast cancer will affect the process of clinical decision making in the daily practice. We are still awaiting results of ongoing clinical trials with gene expression-based prognostic classifiers to eventually implement them in the clinical practice. For now, available predictive models to inform the systemic treatment of individual patients are still limited to a few established biomarkers (hormone receptor and HER2 status, and markers of cell proliferation). On the topic of microenvironment and immune system several points have been addressed. Tumor promoting inflammation is considered to be one of the enabling characteristics of cancer development. Chronic inflammatory disease increases the risk of some cancers and there is strong epidemiological evidence that NSAIDs, particularly aspirin, are powerful chemopreventive agents. Tumour microenvironments contain many inflammatory cells and complex networks of inflammatory mediators such as cytokines and chemokines. Pre-clinical experiments in mouse models have shown that targeting these inflammatory networks with therapeutic antibodies or small molecule inhibitors can decrease tumor growth and spread. While there is exciting potential for targeting inflammatory pathways in cancer prevention, targeting cancer-related inflammation and innate immunity in patients with advanced cancer has not yet proven as promising an approach as recent immunotherapies that modulate the adaptive immune system. A fundamental “dogma” of tumor immunology and of cancer immunosurveillance in particular is that cancer cells express antigens that differentiate them from their non-transformed counterparts. Tumor antigens are overexpressed normal proteins and therefore are subject to immunological tolerance. Immune system controls not only tumor “burden” (quantity) but also tumor “quality” (immunogenicity). As a consequence of constant immune selection pressure placed on genetically unstable tumor cells held in equilibrium, tumor cell variants will be selected. They are no longer recognized by adaptive immunity (antigen loss variants or tumors cells that develop defects in antigen processing or presentation) [10]. They become insensitive to immune effector mechanisms, or induce an immunosuppressive state within the tumor microenvironment (tolerance). These tumor cells may then enter the escape phase, in which their outgrowth is no longer blocked by immunity. Is breast cancer immunogenic? Many observations demonstrated the prognostic role of TILs in TNBC and HER2 positive breast cancer. TNBC are poorly differentiated tumors with high genetic instability and very high heterogeneity. This heterogeneity enhances the “danger signals” and select clone variants that could be more antigenic or, in other words, that could more strongly stimulate a host immune antitumor response. Better prognosis in patients with TN/HER2 positive BC and higher TILs is also the result of an “immunoediting” process induced by chemotherapy. Chemotherapy can stimulate the immune system to recognize and destroy malignant cells, which is commonly known as immunogenic cell death (ICD). Cancer cells succumbing to ICD are de facto converted into an anticancer vaccine and as such elicit an adaptive immune response. What are the clinical implications of all “immunome” data produced in recent years? First, validation of whether TILs are prognostic or predictive in HER2+ and TN breast cancer is needed, preferably in a large population set with appropriate follow up time. Second, validate immune genomic signatures that may be predictive and prognostic in patients with triple negative and HER2 positive disease. Third, it will be essential to incorporate an ‘immunoscore’ into traditional classification of breast cancer, thus providing an essential prognostic and potentially predictive tool in the pathology report. Fourth, implement clinical trials for TN and HER2 positive breast cancer in the metastatic setting with drugs that target immune-cell-intrinsic checkpoints. Blockade of one of these checkpoints, cytotoxic T-lymphocyte-associated antigen 4 (CTLA-4) or the programmed death 1 (PD-1) receptor may provide proof of concepts for the activity of an immune-modulation approach in the treatment of a breast cancer. We need also to better assess the role of TILs in DCIS and to better explore the relationship between autoimmune disease and cancer. The immune system remembers what it targets, so once the system is correctly activated, it may mediate a durable tumor response [10].

## Emerging “drugable” pathways in breast cancer

Dr Finn R.S. discussed about the CDK 4-6 pathway. Cell cycle dysregulation has long been recognized as a “hallmark” of cancer. In normal cells, this complex process is tightly regulated by the interactions of several proteins throughout the various checkpoints and phases (i.e. G1, S, G2, etc). The cyclins and their associated signaling partners, the cyclin-dependent kinases (CDKs), are expressed and function throughout the cell cycle, with specific cyclin–CDK pairs functioning in various phases of the cycle. In G1, CDK 4/6 and cyclin D play a key role in regulating phosphorylation of the RB gene product (pRb). First generation CDK inhibitors did not progress far in clinical development given toxicity and the lack of significant clinical activity. These drugs were generally pan-CDK inhibitors. More recently, highly specific CDK inhibitors have come into development. These include palbociclib (PD-0332991), abemaciclib (LY2835219), and LEE011 all of which have preferential activity against CDK 4/6. All preclinical studies led to the rational design of a Phase I/II study to evaluate the safety and efficacy of palbociclib in combination with letrozole in the treatment of advanced ER+ breast cancer [11]. The PALOMA-1/TRIO 18 study represents a global randomized phase II study that evaluated the combination in two cohorts of women with ER+/HER2− breast cancer: (1) selected for being ER+/HER2− (n = 66) and (2) selected for being ER+/HER2− as well as having the additional biomarkers of cyclin D1 amplification and/or loss of p16 (n = 99) [11]. The results of this study revealed a significant improvement in progression-free survival (PFS) in each of these cohorts. When the entire intent to treat population from both cohorts was analyzed together, PFS increased from 10.2 month to 20.2 months (HR 0.488, 95% CI 0.319–0.748; one-sided p = 0.0004) confirming the preclinical observations that addition of palbociclib to letrozole would improve efficacy in ER+/HER2− breast cancers. This PFS improvement was associated with a manageable side effect profile with the most common adverse events being neutropenia and fatigue. Based on these data, palbociclib was designated a “Breakthrough Therapy” by the US FDA in April of 2013 [11]. Dr Andrew Tutt addressed the DNA repair pathways. A high proportion of breast cancers demonstrate high degrees of genome instability. This genome instability leads to a very large number of copy number aberrations and mutations many of which have low frequency or recurrence across the disease. This genome instability is itself heterogeneous in form resulting from likely variations in DNA repair competency between and within the reported biological groups of breast cancer.

It has long been recognised that there is an association between familial predisposition to breast cancer and the Triple Negative Breast Cancer (TNBC). This is driven by the specific enrichment for TNBC in the breast cancers arising in BRCA1 mutation carriers. Loss of function of BRCA1 or BRCA2 leads to impairment of an accurate DNA repair process called Homologous Recombination (HR) used by proliferating cells to repair DNA replication forks that encounter spontaneous or therapeutic damage in DNA. Failure of HR causes a high degree of genome instability that can have distinctive features driven by the cell’s need to use other DNA repair processes. This leads to high levels of platinum cell kill in preclinical studies of BRCA1 and BRCA2 mutation [12]. Recently the use of PARP inhibitors has been shown to kill malignant cells with deficient HR, such as those with BRCA1 and BRCA2 mutation, through “synthetic lethality”. Dr Trutt reviewed the biological mechanisms relevant to these approaches within specific breast cancer types and discussed some emerging companion diagnostic approaches that seek to identify breast cancers that have deficiencies in HR and might benefit from platinum or DNA repair inhibitor therapies. Dr Baselga Josè discussed about the PIK3CA pathways. Pharmacologic and genetic evidences point to the PI3K/AKT/mTOR pathway as a key mediator of oncogenic signaling in breast cancer. PIK3CA, the gene encoding for p110α, is frequently mutated in human cancers. In particular, hot spot mutations of this gene reside in the helical (E542K and E545K) or catalytic (H1047R) domains are found in over a third of estrogen receptor (ER)-positive breast cancer, representing the most common genomic alteration in this group of tumors [13]. Direct pharmacologic inhibition of the PI3K/AKT/mTOR signaling is, therefore, an attractive clinical strategy for breast cancer. More recently, PI3Kα specific inhibitors have shown remarkable clinical activity in the phase I setting in patients with breast tumors that harbor PI3Kα mutations and these agents are also entering now phase III studies in combination with hormonal therapies. The underlying reason to study these agents in combination with hormonal therapies is compelling. Given that the vast majority of PIK3CA-mutant tumors are ER-positive, it is plausible to hypothesize that both pathways can drive proliferation and survival in these cells. In addition, it is known that anti-estrogen therapy induces the activation of the PI3K pathway in vitro and we have also observed that PI3K inhibition results in a powerful activation of ER signaling. In summary, there is ample evidence that PI3K inhibition will be a fruitful approach in the treatment of patients with advanced breast cancer and it is likely that determining the presence of PI3Kα mutations in breast cancer will become useful in the daily clinical practice. Dr N. Turner discussed the fibroblast growth factor receptor pathway (FGFR). The Fibroblast Growth Factor Receptors can be activated by diverse mechanisms in breast cancer, including amplification of FGFR1 and FGFR2, rare activating mutations and translocations, as well as potentially through aberrant ligand dependent signaling. Amplification of FGFR1 occurs in 10% of ER positive cancer, enriched in luminal B type breast cancer, with FGFR1 amplification associating with increased risk of relapse. A number of early phase clinical trials have selected breast cancers with FGFR1 amplification, providing preliminary evidence of activity for small molecule FGFR inhibitors in FGFR1 amplified breast cancer [14, 15].

## Pathology of breast cancer in 2015

In this session, Dr C. Denkert presented on the role of Ki67 as a prognostic factor. Increased proliferation is a hallmark of malignant tumors and an important parameter for prediction of therapy response. The proliferation marker Ki67 has been suggested as a promising breast cancer biomarker, but the best cutpoints and the best methods for determination are still under debate. The standardisation of Ki67 is still relevant for diagnostic pathology, because this marker can be viewed as the prototype of a quantitative immunohistochemical biomarker, and the experience gained with Ki67 is relevant for other upcoming immunohistochemistry-based prognostic and predictive biomarkers, as well [16]. His presentation gave an overview on the efforts to standardize Ki67 and on the methodological parameters that are relevant for optimal performance of this marker. As for other diagnostic markers, the main parameters for evaluation of Ki67 include (a) clinical validity, (b) analytical validity and (c) clinical utility. Depending on the tumor type and the clinical setting, Ki67 is a mixture of a prognostic and a predictive marker. The combination of both prognostic and predictive effects will determine the performance of Ki67 as a biomarker in different clinical cohorts. Several different cutpoints for Ki67 have been reported to be significant and it is very difficult to determine an evidence-based “optimal” cutpoint. This supports the view that Ki67 should be regarded as a continuous marker, reflecting the continuous variation of the proliferation rate in different types of tumors.

## Primary systemic therapy of breast cancer

### Neoadjuvant chemotherapy

Primary systemic therapy is a standard option for treating patients with early stage breast cancer. Patients achieving a pathological complete response (pCR) have a better disease-free and overall survival. The hazard between pCR and no pCR is largest in TNBC and HER2+ patients [17]. Changing the treatment midway according to clinical response does not influence the long-term outcome, whereas in patients with hormone-receptor positive tumours pCR is important, but the long-term outcome can be improved by adapting the therapy based on clinical response. New studies are currently investigating the role of PARP-inhibitors irrespective of the gene BRCA status. Whereas in HER2+ breast cancer the pCR could be increased to over 70% by using the double HER2 blockade either with trastuzumab plus lapatinib or trastuzumab plus pertuzumab in addition to an 18–24 weeks anthracycline/taxane based chemotherapy. In all trials patients with a HER2+/HR+ tumour had a significantly lower pCR rate than those with a HER2+/HR− tumour. Besides the HR-status, several other biomarkers have been investigated to select patients with the highest chance for a pCR. The PI3Kinase pathway has been investigated by several groups [18]. It could be demonstrated that tumours harbouring a mutation of the PIK3CA have a significantly lower pCR rate with trastuzumab and lapatinib than those with wild-type PIK3CA [18]. However, it is not yet prime time for selecting patients according to the PIK3CA status. Tumour infiltrating lymphocytes and immune markers significantly independently predict a higher pCR rate [19].

### Neoadjuvant endocrine therapy

Neoadjuvant endocrine treatment has become of increasing interest for downstaging primary ER positive breast cancers as it has become clear that the pathologic complete response rate of luminal tumours to chemotherapy is much lower than that of non-luminal and differs little from that from endocrine therapy. There is much more experience in postmenopausal than premenopausal women. Aromatase inhibitors are generally the agent of choice. Responses are lower in those with the low levels of ER. While duration of endocrine treatment in clinical trials has usually been standardized at around three to four months it is clear that volume reductions continue to occur beyond that time in a large proportion of cases and routine clinical practice is often to treat to maximum response. The dependence of responses on the reduced proliferation underpins the value of Ki67 as an intermediate end-point for treatment benefit with multiple studies having found that relative effects on proliferation by different drugs in neoadjuvant trials match their relative impact on recurrence [20].

## Radiotherapy in breast cancer

Dr John Yarnold, Dr Timothy Whelan, and Dr Ian Kunkler addressed the special topic of radiation therapy in early breast cancer. The effectiveness of curative radiotherapy (RT) is enhanced by partitioning the total dose into daily dose increments, called fractions. As a general rule, cancers respond to the total dose more strongly than to the size of daily fractions used to deliver it. This contrasts with the response of late-reacting, dose-limiting healthy tissues, which are sensitive both to the total dose and to the fraction size. These differences underpin the historical use of ‘small’ fractions, classically ≤2.0 Gy, to deliver the highest total dose tolerated by patients, thereby ensuring the greatest impact on cancerous tissue [21]. The general rule has been challenged by randomised clinical trials over the last 20 years providing high level evidence that breast cancer is, against earlier assumptions, equally sensitive to fraction size as the dose-limiting healthy tissues. Theoretical concerns about the effects of dose inhomogeneity are much too small to have any discernible clinical impact, regardless of how RT is fractionated. Full exploitation of differences in fractionation sensitivity between tumours and dose-limiting healthy tissues depends on understanding the underlying molecular mechanisms, and being able to manipulate them appropriately in tumours and dose-limiting normal tissues. In this respect, it has long been understood that DNA is the critical target of therapeutic ionising radiation, and that a critical lesion responsible for cell death is the unrepaired DNA double strand break (DSB). The relevance to fractionation is that we postulate a dominant role for NHEJ and HR in explaining differences in tissue sensitivity to fraction size. The important feature of this model is that it explains the tight association between proliferative indices and sensitivity to fraction size in healthy tissues, which is likely to be affected by extensive epigenetic and epigenetic modifications to DNA damage signalling and repair that are typical of human cancer [21, 22].

## Adjuvant systemic therapy

### Endocrine therapy in premenopausal women

Dr Nancy Davidson addressed in her lecture the topic of endocrine therapy in premenopausal breast cancer women. Multiple strategies for endocrine treatment of premenopausal women with ER positive breast cancer have been assessed and results have been presented over the last two years. Many of these trials have taken place in the backdrop of (neo)adjuvant chemotherapy which can confound interpretation because such therapy can suppress ovarian function either transiently or permanently [5, 6]. The SOFT trial does not show a major advantage for use of OFS + tamoxifen compared to tamoxifen alone [6]. The joint SOFT/TEXT analysis and ABCGS12 trials both suggest that outcomes can be excellent with the use of combined endocrine therapy alone in properly selected patients but give conflicting results with regard to potential benefits for OFS + AI compared with OFS + tamoxifen [7]. Given the long natural history of endocrine-responsive breast cancer, longer follow-up of all trials will be critical to ascertain benefit and document unexpected late toxicities, if any. A pressing need is the identification of markers that can identify patients who need extended adjuvant endocrine therapy as well as markers that might allow selection of those that would benefit from a combination strategy like OFS + AI. What is clear from all these studies is that adjuvant endocrine therapy is a vital part of the adjuvant regimen for most premenopausal women with hormone-responsive breast cancer and a subset of these women with luminal A-type tumors can be safely treated with endocrine therapy alone.

### Endocrine therapy for postmenopausal women

Dr Harold Burstein addressed the topic of endocrine therapy in postmenopausal women. Treatment options for postmenopausal women include either tamoxifen (Tam) or an aromatase inhibitor (AI) or a sequence of the two, given for 5 or 10 years. Both can cause menopausal symptoms such as hot flashes and night sweats. Tamoxifen contributes to rare risks of uterine cancer and thromboembolism. AIs cause accelerated osteoporosis and bone fracture, a musculoskeletal syndrome associated with achiness and stiffness, and vaginal atrophy with associated sexual dysfunction. In comparison to 5 years of tamoxifen monotherapy, the incorporation of an AI into the treatment program either as initial treatment, or as sequential therapy after 2–3 years of tamoxifen, lowers the risk of cancer recurrence. Patient preferences factoring in side effect profiles and individual tolerability are important in choosing one strategy or the other. Extended adjuvant endocrine therapy further reduces the risk of recurrence. Longer durations of treatment improve on outcomes seen with TAM alone, but also carry risks of ongoing side effects. There are no data for the safety or efficacy of AI therapy beyond a total duration of 5 years though clinical experience does not suggest emerging toxicity concerns. Predictors of recurrence in ER positive breast cancer include tumor and nodal stage at presentation, tumor grade, measures of proliferation such as Ki67, quantitative degrees of ER/PR expression, intrinsic subtype, and molecular diagnostic profiles. These pathobiological variables are highly interrelated across a spectrum. Tumors that are low grade typically have high degrees of ER and PR expression, lower proliferation measures, lower OncotypeDX recurrence scores, and tend to cluster as luminal A tumors. To date, there are no markers that selectively predict early vs late recurrence, nor suggest treatment with tamoxifen vs AI, nor determine who benefits from longer duration of therapy. Almost all women diagnosed with ER positive tumors should consider endocrine therapy. Even subcentimeter, node-negative cancers have a lower risk of recurrence with use of endocrine treatment.

## Adjuvant chemotherapy in early breast cancer

### Triple negative breast cancer

De Eric Winer presented an overview on the treatment of triple negative early breast cancer. Triple negative breast cancer accounts for approximately 10–15% of all breast cancer and may be responsible for a higher proportion of cases in developing countries. By definition, all triple negative cancers do not have estrogen and progesterone receptors and they are not amplified for HER2. As a result, the only available adjuvant treatment consists of chemotherapy. For patients with stage II and III triple negative disease, regimens that contain anthracyclines and taxanes are the standard approach. Those with stage I disease are often treated with shorter and/or somewhat less toxic regimens. In general, chemotherapy reduces the overall risk of disease recurrence in the triple negative setting by as much as 35–50%. In spite of the impact of chemotherapy, triple negative disease accounts for a disproportionate share of breast cancer mortality. Some triple negative cancers are exquisitely sensitive to chemotherapy and others have a high degree of intrinsic resistance to the same therapy. It has become clear that triple negative disease is remarkably heterogeneous. While almost all triple negative cancers are poorly differentiated have a high degree of genomic instability, there are important different across triple negative tumors. A variety of investigators have attempted to sub-classify triple negative cancers, and there do appear to be substantial differences in the genetic make-up of triple negative cancer, but the clinical significance of these genomic sub-classifications remain unclear. The subgroup of triple negative cancers that have a high proportion of tumor infiltrating lymphocytes (TILS), have a better overall prognosis, probably in part because of an increased sensitivity to cytotoxic chemotherapy. A number of investigators are examining the subgroup of tumors that have a defect in homologous recombination, a feature of BRCA1-associated triple negative cancers. Several studies are attempting to determine if these tumors are more sensitive to platinum-based therapy. At present, we cannot use investigational classifications of triple negative breast cancer to determine appropriate systemic therapy. These approaches are in the early phases of investigation in clinical trials. While it is hoped that one or more sub-classifiers will be useful in the clinical setting, we will have to wait at least 2–3 years before utilizing these approaches outside of clinical trials.

### HER2 positive breast cancer

Dr Martine Piccart-Gebhart addressed the HER2 positive disease. Recently, two additional HER2 blocking agents became part of our therapeutic armamentarium: trastuzumab-DM1, an antibody-drug conjugate, delivering potent cytotoxic chemotherapy to HER2-overexpressing cancer cells, while retaining the biologic actions of trastuzumab; and pertuzumab, an anti-HER2 monoclonal antibody, which when coupled with trastuzumab achieves a more thorough HER2 blockade, capitalizing on the concept of dual HER2 blockade [23, 24]. Despite these advances, patients with HER2-positive BC still experience relapse of their disease, with the resistance being almost inevitable in the metastatic stage. To this end, currently ongoing research efforts try to further improve the clinical outcomes within this patient population, focusing on the following areas: (i) assessment of different dual HER2 blockade strategies, (ii) development of TDM1 in the early stage disease, (iii) co-targeting of HER2 with other molecules perceived as anti-HER2 treatment resistance mediators, and (iv) identification of predictive biomarkers. Numerous other potentially relevant alterations are being assessed as predictive biomarkers for HER2 blockade, with extensive molecular profiling initiatives currently ongoing. These are particularly important in view of the recent negative results of the large ALTTO trial which tested 4 different anti-HER2 in combination or in sequence with adjuvant chemotherapy: trastuzumab or lapatinib alone, the sequence and the combination of the 2 agents. In the era of personalized oncology, rigorous translational and clinical collaborative efforts are needed to further advance the field of treatment of patients with HER2-positive breast cancer.

## Treatment of special patient populations

### Very young patients

Dr Ann Partridge focused on the treatment of very young patients. Breast cancer is the leading cause of cancer-related deaths in women age 40 and younger in developed countries, and although generally improving, survival rates for young women with breast cancer remain lower than for older women. Young women are more likely to develop more aggressive subtypes of breast cancer (more triple negative and HER2-positive disease) and present with more advanced stage disease. Previous research has demonstrated that young age is an independent risk factor for disease recurrence and death, although recent data suggest this may not be the case in certain tumor molecular subtypes. Recent preliminary evidence suggests potential unique biologic features of breast cancer that occurs in young women although this has yet to have been translated into treatment differences. There are clearly host differences that affect the management of breast cancer for young patients including generally being very premenopausal at diagnosis, and concerns regarding fertility, genetics, and social/ emotional issues that should be considered. Despite an increased risk of local recurrence, young age alone is not a contraindication to breast conserving therapy given the equivalent survival seen in this population with either mastectomy or breast conservation. However, many young women in recent years are choosing bilateral mastectomy, even without a known hereditary predisposition to the disease. For systemic therapy, endocrine therapy, multi-agent chemotherapy and/or biologic therapy decisions should target the tumor similar to the treatment in older women. Attention to adherence with endocrine therapy may be particularly important to improve outcomes in young survivors who are at increased risk of non-adherence compared to older women.

### Elderly patients

Dr Ian Smith focused on treatment of elderly patients. The incidence of breast cancer continues to rise, particularly in the elderly. The median life expectancy of a 70 year old European woman is 16 years and an 80 year old almost 10 years. The challenge in older patients is to balance effective treatment against potential comorbidities and to minimize both under-treatment and over-treatment. An additional important issue is that older patients are under-represented in clinical trials, such that a strong evidence base for therapeutic decisions is often lacking. Avoidance of, or minimizing, surgery simply because of age is bad management: trials have shown that tamoxifen alone in women over 70 is associated with both increased risk of local recurrence and worse survival than with surgery as well. Likewise adjuvant chemotherapy should not be withheld on the basis of age alone. Patient selection is however as important as in other age groups. Treatment related mortality however is higher in women over 65 (1.5%) compared with those <50 (0.2%). Balanced against this, conventional chemotherapy in the elderly is more effective than less toxic single agent oral treatment. Further trials of less toxic and simpler chemotherapy in the elderly are urgently needed. The assessment of comorbitities, general frailties and their prognostic influence is therefore important. Measures available include Comprehensive Geriatric Assessment (CGA) and Charlston index are available to assess prognosis independent of cancer and fitness for treatment. In contrast to surgery and chemotherapy, post surgical radiotherapy may be overused in older women. Many of the studies on which this practice is based excluded patients aged 70 or older, and older age is a recognised factor predicting a lower risk of local recurrence following breast conserving surgery. Randomised trials strongly suggest that older women with small breast cancers on tamoxifen after surgery may gain little or nothing from adjuvant radiotherapy. In conclusion, further trials of less toxic/less morbid therapeutic approaches that still sustain efficacy are now urgently needed for older/frail patients, with the long term hope that these might subsequently prove useful in younger fitter patients as well.

## Conclusions

The St Gallen Consensus Conference is designed to ask questions about controversial issues. This is the unique feature of the St. Gallen Consensus, ensuring that the resulting recommendations will take due cognizance of the variable resource limitations in different countries. Information derived from evidence based medicine and large meta-analyses is of obvious and enormous value. The weakness of this approach is that it gives particular weight to older trials (which have accumulated more event endpoints) and is frequently unable to collect sufficient detail on the patients and tumors in the trials to allow assessment of whether the treatments which are better on average offer equal value to all currently definable patient subgroups. What St Gallen can provide is clinically useful updated breast cancer treatment consensus for the majority of patients treated outside of clinical trials (>90%) in most countries.
